# Biotinylated-sortase self-cleavage purification (BISOP) method for cell-free produced proteins

**DOI:** 10.1186/1472-6750-10-42

**Published:** 2010-06-04

**Authors:** Satoko Matsunaga, Kazuhiro Matsuoka, Kouhei Shimizu, Yaeta Endo, Tatsuya Sawasaki

**Affiliations:** 1 The Cell-Free Science and Technology Research Center, Ehime University, 3 Bunkyo-cho, Matsuyama, Ehime 790-8577, Japan; 2 The Venture Business Laboratory, Ehime University, 3 Bunkyo-cho, Matsuyama, Ehime 790-8577, Japan; 3 Proteo-Medicine Research Center, Ehime University, Toon, Ehime 791-0295, Japan; 4 RIKEN Systems and Structural Biology Center, 1-7-22 Suehiro-cho, Tsurumi, Yokohama 230-0045, Japan

## Abstract

**Background:**

Technology used for the purification of recombinant proteins is a key issue for the biochemical and structural analyses of proteins. In general, affinity tags, such as glutathione-S-transferase or six-histidines, are used to purify recombinant proteins. Since such affinity tags often interfere negatively with the structural and functional analyses of proteins, they are usually removed by treatment with proteases. Previously, Dr. H. Mao reported self-cleavage purification of a target protein by fusing the sortase protein to its N-terminal end, and subsequently obtained tag-free recombinant protein following expression in *Escherichia coli*. This method, however, is yet to be applied to the cell-free based protein production.

**Results:**

The histidine tag-based self-cleavage method for purifying proteins produced by the wheat cell-free protein synthesis system showed high background, low recovery, and unexpected cleavage between the N-terminally fused sortase and target protein during the protein synthesis. Addition of calcium chelator BAPTA to the cell-free reaction inhibited the cleavage. In order to adapt the sortase-based purification method to the cell-free system, we next used biotin as the affinity tag. The biotinylated sortase self-cleavage purification (BISOP) method provided tag-free, highly purified proteins due to improved recovery of proteins from the resin. The N-terminal sequence analysis of the GFP produced by the BISOP method revealed that the cleavage indeed occurred at the right cleavage site. Using this method, we also successfully purified the E2 heterocomplex of USE2N and USE2v1. The c-terminal src kinase (CSK) obtained by the BISOP method showed high activity in phosphorylating the Src protein. Furthermore, we demonstrated that this method is suitable for automatically synthesizing and purifying proteins using robots.

**Conclusion:**

We demonstrated that the newly developed BISOP method is very useful for obtaining high quality, tag-free recombinant proteins, produced using the cell-free system, for biochemical and structural analyses.

## Background

Technology used for purifying a recombinant protein has a significant impact on its biochemical function, structural properties, and other aspects, such as generating an antibody against the protein. Currently available, established purification methods generally attach an affinity tag to the N-terminus or C-terminus end of the target protein, and then recover the target protein by affinity chromatography [1]. Purification tags used today are classified into peptide-tags and protein-tags based on their nature and form. His-tag, a typical and globally the most used peptide-tag, is highly effective in purifying the tagged protein by using immobilized metal affinity chromatography, such as nickel sepharose [2]. The glutathione-S-transferase (GST) tag, a typical protein-tag, has a high specific binding capacity for glutathione, and is, generally, known to have little or no effect on the activity of the fused recombinant protein [3]. In both cases, however, production of tag-free recombinant protein requires treatment with a protease, such as PreScission or TEV protease. Therefore, in order to purify a tag-free recombinant protein multiple chromatography steps are necessary.

Currently, there are only a few recombinant protein purification methods that combine affinity purification, cleavage, and separation of the fusion partner in one-step. One such one-step purification method was reported by Mao [4], in which the catalytic core of the transpeptidase sortase A (srtA, amino acid residues from 60-206), found in the cell envelope of *Staphylococcus aureus *[5,6], was used for fusion with the target protein. The SrtA enzyme is known to catalytically cleave the Thr-Gly bond of its recognition motif LPXTG (X is any amino acid) in the presence of calcium and triglycine [6-9]. Thus, the purified target protein eluted off the affinity column has only an extra Gly residue on the N-terminus end. This excellent approach was designed for the purification of recombinant proteins expressed in *Escherichia coli *cells. However, as discussed in that report [4], the fusion protein was partially self-cleaved during the expression, probably because of the difficulty in controlling the concentration of calcium in the living cells. Inability to suppress the srtA activity during the expression of the fused recombinant protein in *E. coli *was, therefore, a major limiting factor for using this otherwise excellent approach as a general tool for the production and purification of recombinant proteins.

At present, several types of cell-free protein production systems have been reported as alternative methods for obtaining recombinant proteins [see 10 and 11 for reviews]. In this regard, it is noteworthy that we are also developing wheat embryo based cell-free system for in vitro protein production [12-14]. Cell-free protein production is very flexible because it utilizes only the translational machinery of the cell without other factors, such as DNA replication and metabolic pathways, of the living system. The cell-free system, thus, could simply be modified by the addition or subtraction of reagents. In this study, we have adopted the self-cleavage activity of srtA in the wheat cell-free system for the production of tag-free recombinant proteins, and demonstrated an improved self-cleavage purification method by incorporating biotinylation reagents and calcium chelates in the cell-free synthesis system.

## Results and Discussion

### Self-cleavage activity of srtA during the cell-free protein synthesis

**Figure 1:**
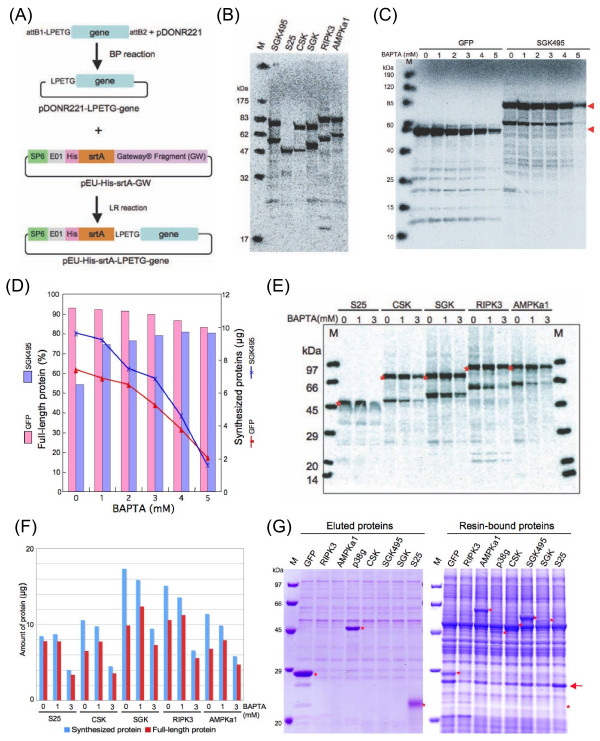
**Synthesis of srtA-fusion proteins using the wheat germ cell-free system**. **A**. Schematic representation of the pEU-His-srtA-LPETG-Gene plasmids created using the Gateway system. **B**. Autoradiogram of SDS-PAGE of proteins synthesized using the cell-free system in the presence of [^14^C] Leu. Lane M, Protein MW standards labeled by using [^14^C]-containing felt pen. **C**. Autoradiogram of [^14^C] Leu incorporated GFP and SGK495 proteins synthesized by the wheat cell-free system in the presence of the Ca^2^+ chelating reagent BAPTA. The number represents concentration (mM) of BAPTA used in the protein synthesis reaction. Arrowheads denote the sizes of the full-length proteins. **D**. Rate of synthesis of the full-length protein and productivity of GFP (pink-colored bar and red-colored line) and SGK495 (purple-colored bar and blue-colored line) in the presence of different concentrations of BAPTA. **E**. Autoradiogram of [^14^C]-Leu incorporated proteins synthesized by the cell-free system in the presence of BAPTA. Asterisk denotes the sizes of the full-length proteins. **F**. Rate of synthesis of the full-length protein and productivity of proteins in the presence of different concentrations of BAPTA. Productivities of total synthesized and full-length proteins indicated as blue and red bars respectively. **G**. Purifications of proteins by the cell-free synthesis using the pEU-His-srtA-LPETG-Gene plasmid constructs. CBB-stained protein bands on the SDS-PAGE gel of the eluted (left panel) and resin-bound (right) target proteins are indicated using asterisk. Arrow represents the cleaved His-tagged srtA. Lane M (both panels): Protein MW standards.

First, based on the previous report, we constructed the expression vector pEU-His-srtA-GW by inserting the DNA fragment required for the Gateway (GW) recombination technology into the previously described wheat germ cell-free expression vector pEU-E01 [13]. The Gateway system allows easy recombination of the targeted genes. The srtA cleavage site, LPETG, was introduced in the forward PCR primer according to the instructions provided with the Gateway system (Figure 1A). To test the self-cleavage purification system, we selected the human protein kinases and malaria vaccine candidate Pfs25 *(Plasmodium falciparum *25 kDa ookinete surface antigen precursor) as candidate proteins because they are very important proteins for practical use. PCR products of the coding regions of Pfs25 and six protein kinases were amplified, and each PCR amplified fragment was individually inserted into the pDONR221 vector by BP recombination reaction. Subsequently, the inserted LPETG-gene fragment was cloned into the pEU-His-srtA-GW vector by LR recombination reaction, resulting in a pEU-His-srtA-LPETG-gene plasmid. These pEU-His-srtA-LPETG-gene plasmids were then used for ^14^C-Leu-labeled protein synthesis using the wheat cell-free system. Unfortunately, in all cases, 20 to 40% of the synthesized proteins were cleaved during the cell-free synthesis (Figure 1B). The cleavage rate was dependent on the type of the gene used in creating the plasmid construct. For example, cleavage of Pfs25 (S25 lane in Figure 1B) and GFP (Figure 1C) during the protein synthesis process were very low, whereas almost 40% of the synthesized SGK495 protein was cleaved during the cell-free synthesis. Since calcium supplementation is known to induce the srtA activity [4,9], the cell-free system was treated with the calcium chelator, BAPTA (Figure 1C). Treatment with more than 4 mM BAPTA dramatically blocked the inexpedient cleavage of SGK495. However, protein synthesis was inhibited as the BAPTA concentration was increased (Figure 1D). For example, levels of SGK495 and GFP proteins produced in the presence of 5 mM BAPTA were approximately 20% and 30%, respectively, of their respective levels in the absence of BAPTA. In other proteins, the synthesis conditions in the presence of 1 and 3 mM BAPTA were investigated (Figure 1E and 1F). By comparing the results of the BAPTA-concentration dependent blocking of the srtA activity and inhibition of the protein synthesis, we concluded that 1 mM BAPTA is optimal for the cell-free synthesis of srtA-fusion proteins, because at this concentration of BAPTA the recovery of all full-length srtA fusion proteins improved by approximately 10-20% without any major inhibition of the protein synthesis.

### Self-cleavage purification of His-srtA-fusion proteins

A total of eight plasmid constructs, each containing a different gene (generalized here as pEU-His-srtA-LPETG-gene), were used for the cell-free protein production and self-cleavage purification studies, and the results are shown in Figure 1G. Out of eight proteins, three proteins, GFP, p38g and Pfs25, clearly eluted from a nickel-nitrilotriacetic acid (Ni-NTA) sepharose column, whereas other five proteins could not be recovered in the eluted fraction (Figure 1G, left panel). To confirm protein synthesis and self-cleavage, column resins of all samples were analyzed by SDS-PAGE after boiling with SDS-sample buffer (Figure 1G, right panel). Surprisingly, even though AMPKa1, CSK, SGK and SGK495 were synthesized and self-cleaved on the column resin, their cleaved forms were not eluted off the resin. We could not find the RIPK3 protein in the eluted or in the resin-bound fraction, suggesting that this protein was expressed at a very low level. In addition, purity of the protein in the eluted fraction was not high, as there were several similar protein contaminants in every lane. Furthermore, many proteins bound to the Ni-NTA resin (right panel in Figure 1G). These results suggested that further technical improvements were necessary to achieve high quality purified proteins with high-efficiency from the cell-free based His-srtA system.

### Biotinylated sortase self-cleavage purification (BISOP)

**Figure 2:**
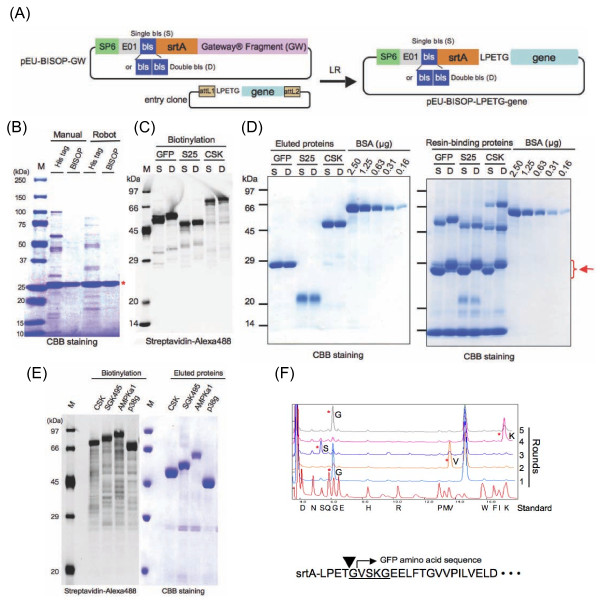
**Preparation of proteins by the biotinylated-sortase self-cleavage purification (BISOP) method**. **A**. Scheme for creating the pEU-BISOP-LPETG-gene plasmids using the Gateway recombination method. The LPETG-fused target gene containing entry clone plasmid was recombined with the pEU-BISOP-GW vector by using the Gateway LR reaction. **B**. CBB-stained SDS-PAGE comparing the His- or biotin-tagged GFP protein (indicated using an asterisk) purified manually or by using an automated robot. Lane M: Protein MW standards. **C**. Biotinylation of GFP, Pfs25 (S25) and CSK proteins synthesized by the cell-free system with vectors having a single (S) or a double (D) biotin ligation site. Biotinylated proteins were detected by labeling the separated protein bands with streaptavidin-Alexa488 as described in the text, followed by scanning using the Typhoon Imager. **D**. CBB-stained eluted (left panel) and resin-bound (right panel) proteins. Arrow indicates the cleaved biotinylated srtA protease left on the resin. Samples (5 or 10 μL in left or right panel respectively) were loaded on the gel. **E**. Left panel: Biotinylated proteins were detected by streaptavidin-Alexa488; Right panel: CBB-stained proteins purified by the BISOP method. Samples (5 μL) were loaded on the gel. **F**. Amino acid sequences from the N-terminal end of GFP purified by the BISOP method were determined by using an amino acid sequencer (asterisks, upper panel). Rounds indicate the number of Edman degradation cycle. Underlined amino acid sequence in the lower panel shows the determined sequence. Arrowhead indicates the site cleaved by the sortase enzyme.

Recently we successfully adapted the biotin-labeling system of *E. coli *biotin ligase and biotin to the wheat cell-free protein synthesis system [15]. The biotinylation reaction modifies a specific lysine residue at the biotin ligation site (bls: GLNDIFEAQKIEWHE, the underline is the ligation site). The biotinylated proteins could be directly used for an assay without further purification because of very low biotin concentration. Since the His-tag based approach showed many contaminated proteins in the eluted fraction of the Ni-NTA column, we next used the biotin-labeled tag for protein purification. For this purpose, we constructed the pEU-BISOP-LPETG-GFP plasmid based on the pEU-His-srtA-LPETG-GFP vector as shown in Figure 1A and 2A. Next, we compared whether the His-tagged or the biotin-tagged protein could be better purified by the sortase self-cleavage method, processed either manually or using automated robots to eliminate any human error. Clearly, on the CBB-stained SDS-PAGE, a single major protein band (with low background) was found in the eluted fraction following the sortase self-cleavage of the biotin-tagged srtA fusion protein; in contrast, contaminating proteins were found along with the major protein band in the eluted fraction of the self-cleaved His-tagged srtA fusion protein (Figure 2B). Reaction performance of robot for His-srtA-LPETG-GFP or BISOP-LPETG-GFP was 63 or 58% of recovery, 52 or 88% of purity, and 68 or 62 μg of yield respectively. These results suggest that the BISOP method is better suited for producing tag-free purified proteins by the cell-free system.

Next, to examine whether the number of bls has any effect on the purification, two vectors, one having a single bls and the other having double bls, were constructed (Figure 2A). Coding regions of GFP, Pfs25 and CSK were individually cloned into each one of these two vectors, and then the resultant recombinant plasmids were used for the protein synthesis using the cell-free system. Staining with Alexa488-labeled streptavidin revealed slight mobility shifts for proteins containing double bls, as compared to those containing single bls (Figure 2C). We did not observe any difference between the single and double bls containing proteins with respect to biotinylation, elution and resin binding characteristics. Similar to the self-cleavage purification of GFP (shown in Figure 2B), all three eluted proteins were highly purified (left panel in Figure 2D). The higher purity of proteins obtained using the BISOP method might be due to the presence of very few contaminating proteins on the streptavidin-conjugated resin (compare right panel in Figure 2D with Figure 1G). Interestingly, the CSK protein, prepared by the BISOP method was eluted of the resin, whereas with the His-tag based method it was not found in the eluted fraction (Figure 1G). Total amounts of purified GFP, S25 and CSK by the BISOP method were 44, 37, and 55 μg per reaction respectively. Also full-length GFP, S25 and CSK proteins remaining on the column were approximately 6, 3 and 3 μg respectively, and cleaved S25 and CSK proteins on the column were 3 and 3 μg respectively. These data means that rate of target proteins remaining on the column was approximately 15%. Next, the BISOP method was used for purifying several other proteins. For this purpose, four protein kinase genes were individually inserted into the Gateway system vector pEU-BISOP-GW following the procedures described above, and the results are shown in Figure 2E. Both SGK495 and AMPKa1, which were not recovered earlier from the resin when the His-tag based method was used (Fgiure 1G), were also purified by the BISOP method (Figure 2E).

Total amounts of purified CSK, SGK495, AMPKa1 and p38g by the BISOP method were 55, 42, 24 and 57 μg per reaction respectively. In addition, analysis of the N-terminal sequence of the GFP protein purified by the BISOP revealed the expected cleavage of the Thr-Gly bond of the inserted LPETG sortase-recognition site (Figure 2F). These results suggested that the BISOP method would be suitable for the purification of the cell-free produced proteins with high efficiency and purity.

### Purification of E2 heterocomplex by BISOP

**Figure 3:**
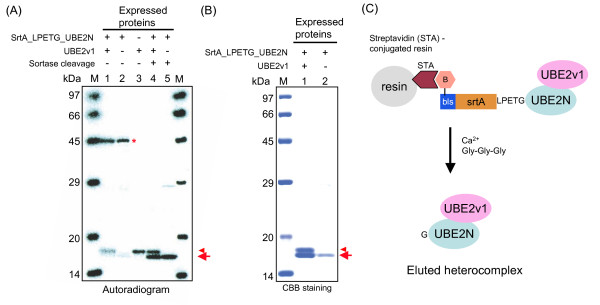
**Application of BISOP method to purify the UBE2N/UBE2v1 heterocomplex co-expressed by the cell-free system**. **A**. The proteins were synthesized from the plasmid constructs using the cell-free system in the presence of ^14^C-Leu and the synthesized proteins were detected by autoradiography after electrophoresis on a SDS-PAGE (5-20% gradient gel). Lane 1: Proteins synthesized from a mixture of pEU-BISOP-LPETG-UBE2N and pEU-UBE2v1 plasmids; Lane 2: Protein synthesized using the pEU-BISOP-LPETG-UBE2N plasmid; Lane 3: Protein synthesized using the pEU-UBE2v1 plasmid; Lane 4: Sortase cleavage of lane 1 by the BISOP method; Lane 5: Sortase cleavage of lane 2 by the method; and Lane M, Protein MW standards. Asterisk, arrow and arrowhead indicate srtA-LPETG-UBE2N, G-UBE2N and UBE2v1 (tag-free), respectively. **B**. BISOP was performed after expression of biotinylated srtA-LPETG-UBE2N with (lane 1) or without UBE2v1 (lane 2). The eluted proteins were separated on the SDS-PAGE gel and then stained with CBB. Arrow and arrowhead indicate the purified G-UBE2N and UBE2v1, respectively. **C**. Scheme for the preparation of UBE2N/UBE2v1 heterocomplex by BISOP.

Analysis of protein complex is one of important targets for their structural and biochemical analysis. Thus, next we examined whether a protein heterocomplex, co-expressed using the wheat cell-free system, could be purified by the BISOP method. To test this notion, we next co-expressed UBE2N and UBE2v1, two proteins forming the heterodimer complex of the ubiquitin-conjugate (E2) enzyme [16], using the BISOP method. Specific complex formation between these two proteins produced by the cell-free system has already been reported [15]. At first, we confirmed co-expression of the biotinylated srtA-LPETG-UBE2N and UBE2v1 (tag-free form) by incorporating ^14^C-Leu during the cell-free synthesis (Figure 3A). Notably, we recovered the E2 heterocomplex consisting of UBE2N and UBE2v1 when both UBE2v1 and srtA-LPETG-UBE2N were co-expressed using the cell-free system and then purified by the BISOP method (Figures 3B and 3C). The protein band corresponding to UBE2v1 was however not found when the biotinylated srtA-LPETG-UBE2N was expressed alone. Therefore, this result suggests that the BISOP method would be useful for purification of protein complexes produced by the cell-free system.

### Activity of CSK purified by the BISOP method

**Figure 4:**
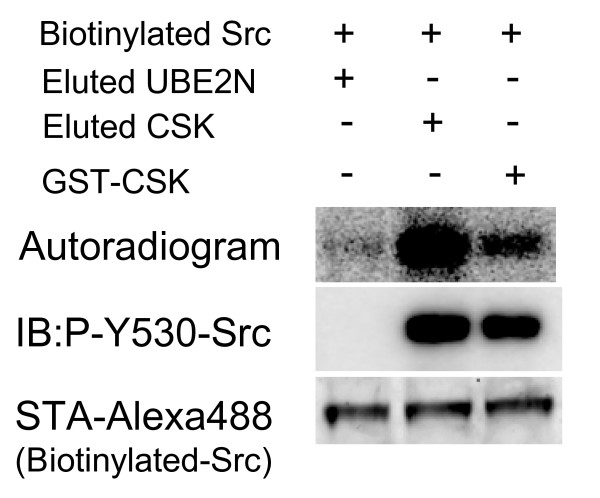
**Activity of CSK prepared using the BISOP method**. Biotinylated Src protein was incubated with UBE2N, CSK purified using the BISOP method (eluted CSK and UBE2N), or commercially available GST-CSK fusion protein in the presence of cold (for immunoblot) or radioisotope-labeled (for autoradiogram) ATP. Src phosphorylation was assayed by immunoblot analysis using the anti-Phospho-Src (Y530) antibody. Autoradiogram shows phosphorylation of the Src protein by CSK.

It is very important that the purification method provide functionally active protein. We, therefore, investigated whether the CSK protein purified using the BISOP method could specifically phosphorylate the Tyr-530 residue of human Src protein (Swiss-Prot no. P12931) [17]. For this purpose, biotinylated Src was synthesized by the cell-free system and the synthesized protein was partially purified using the magnetic streptavidin-conjugated beads. The bead-bound Src was then treated with [^32^P]-labeled or unlabeled ATP and the CSK protein that was purified by the BISOP method. Both autoradiogram of the SDS-PAGE separated proteins from the reaction mix containing [^32^P]-labeled ATP and immunoblot analysis of the SDS-PAGE separated proteins from the reaction mix containing the unlabeled ATP using the specific anti-phospho-Src antibody showed specific phosphorylation of the Y530 residue of Src by CSK (Figure 4). These results suggest that BISOP would be suitable for the in vitro synthesis of active proteins.

## Conclusion

The cell-free system is an easy to use method for synthesizing recombinant proteins. In this study, we have developed a new biotinylated-sortase self-cleavage purification (BISOP) method to achieve high quality purification of several proteins, including a protein heterocomplex, without any affinity tag. In addition, we showed that this method could be automated using robots. Results of this study indicate that the combination of the cell-free protein synthesis system and BISOP could provide a simple and easy method for the preparation of high quality recombinant proteins without any affinity tag. Since the cell-free system is suitable for high-throughput protein production, this combined method could also be utilized at the genome-wide level.

## Methods

### General

The following procedures have been either described in detail or cited [13,15,18,19]: isolation of the wheat germs and preparation of the extract, generation of the DNA template by polymerase chain reaction (PCR) using split-primers, parallel synthesis of mRNA and protein, estimation of the amount of protein synthesized by densitometric scanning of the Coomassie brilliant blue (CBB)-stained band and autoradiogram of radiolabeled-proteins, and detection of biotinylated proteins using Alexa488-conjugated streptavidin (Invitrogen) and the Typhoon Imager (GE Healthcare) fitted with 532 nm laser and 526 emission filter.

### Template genes

The cDNA clone of the malarial parasite *Plasmodium falciparum *25 kDa ookinete surface antigen precursor (Pfs25) was kindly provided by Dr. Tsuboi (Cell-free Research and Technology Center, Ehime University). cDNAs of GFP, UBE2N (GenBank accession no. BC003365), and UBE2v1 (GenBank accession no. BC000468) were described in our previous reports [15,18]. Mammalian gene collection (MGC) cDNA clones of CSK (BC104875), SGK (BC001263), SGK495 (BC007835), AMPKa1 (BC048980), p38g (BC015741), RIPK3 (BC062584) and Src (BC011566) were also used in this study.

### Construction of the srtA-based self-cleavage vector

DNA fragment encoding the mature-form of srtA (corresponding to amino acids 60-206, GenBank accession no. AF162687) was artificially synthesized and inserted into the pUC57 to create the plasmid pUC57-srtA-EcoRV-SpeI by the GenScript Corporation (Boston). The pUC57-srtA-EcoRV-SpeI plasmid as used as a template to amplify the mature srtA fragment (DraI-His or bls-srtA-EcoRV-SpeI) by PCR using the following pair of primers: M13(M3) (5'-GTAAAACGACGGCCAGT) and DraI-A-His-srtA (5'-GAGATTTAAATGGCCAGCAGCCATCACCATCACCATCATAGCAGCGGCCTGGTGCCGC) or M13(M3) and Dra1-A-bls-srtA (5'-GAGATTTAAATGGCCAGCAGCGGCCTGAACGACATCTTCGAGGCCCAGA AGATCGAGTGGCACGAAAGCAGCGGCCTGGTGCCGC). The DraI-A-His-srtA primer included 6 × His-tag codons and a DraI restriction enzyme site, and the DraI-A-bls-srtA primer included a bls (biotin ligase site) recognition sequence and a DraI restriction enzyme site. After digestion with DraI and SpeI enzymes each fragment was inserted into the EcoRV and SpeI sites in pEU-E01-MCS provided by CellFree Sciences, Ltd http://www.cfsciences.com/eg/index.html to create pEU-His-srtA-MCS and pEU-BISOP-MCS plasmids, respectively. To create the Gateway^®^-based plasmids pEU-His-srtA-GW and pEU-BISOP-GW, DNA fragment needed for Gateway^® ^recombination cloning technology (Invitorogen) was inserted into the EcoRV site of the pEU-His-srtA-MCS and pEU-BISOP-MCS plasmids. PCR reaction was performed by PrimeStar enzyme according to instruction (Takara Bio, Otsu, Japan). Nucleotide sequences of the DNA inserts in all plasmid constructs were subsequently confirmed by using the ABI PRISM 310 Genetic Analyzer using the BigDye terminator v1.1 Cycle sequence kit (Applied Biosystems, Foster City, CA).

### Plasmid construction for the cell-free protein production

We introduced the srtA self-cleavage site (DNA encoding for the amino acid sequence LPETG) into the recombinant plasmid construct for the cell-free production of proteins. The DNA fragments coding the respective protein were amplified by PCR using two gene specific primers: forward primer attB1-LPETG-Gene (5'-GGGGACAAGTTTGTACAAAAAAGCAGGCTTC**CTGCCCGAGACCGGC**atg(n)_19_; uppercase, lowercased and bold sequences indicated common, gene specific and LPETG sequences, respectively; n represent gene specific sequence) and reverse primer attB2-Gene (5'-GGGGACCACTTTGTACAAGAAAGCTGGGTCxxxnnnnnnnnnnnnnnnn; xxx is the complementary sequence of the stop codon; n represent gene specific sequence). PCR reaction was performed using the PrimeStar enzyme (Takara Bio, Otsu, Japan) and following the supplier's instructions. The amplified attB1-LPETG-Gene-attB2 fragments were inserted into the donor vector pDONR221 by BP reaction to generate the entry plasmids. The LPETG-fusion Gene in the entry plasmid was transferred to the pEU-His-srtA-GW or pEU-BISOP-GW by LR reaction to generate the pEU based-plasmid clones. BP and LR reactions were carried out according to the instructions provided by the supplier of the reagents (Invitrogen, Carlsbad, CA). Nucleotide sequences of the DNA inserts in all the plasmid constructs were subsequently confirmed by using the ABI PRISM 310 Genetic Analyzer described method above.

### Cell-free protein production

For the cell-free protein production, we employed the wheat germ cell-free protein expression system using the bilayer translation method described previously [18,19]. Cell-free protein production was carried out using the ENDEXT^® ^Wheat Germ Expression S Kit and according to the instructions provided by the supplier (CellFree Sciences Co., Ltd., Matsuyama, Japan). Briefly, 250 μL of transcriptional mixture [80 mM HEPES-KOH, pH 7.8, 16 mM magnesium acetate, 2 mM spermidine, 10 mM DTT, 2.5 mM NTP mix, 1 U/μL SP6 RNA polymerase (Promega, Madison, WI), 1 U/μL RNase inhibitor, RNasin (Promega), and 100 ng/μL undigested plasmid DNA] was incubated at 37°C for 6 h, and then mixed with 250 μL of wheat embryo extract (120 A 260/mL, CellFree Sciences Co., Ltd.) and 1 μL of 20 mg/mL creatine kinase (Roche Applied Science, Indianapolis, IN). This mixture, called the translational mixture, was then carefully transferred to the bottom of the well of a 6-well tissue culture plate (Whatman Inc., Clifton, NJ) that already contained 5.5 mL of TSB (30 mM HEPES-KOH, pH 7.8, 100 mM potassium acetate, 2.7 mM magnesium acetate, 0.4 mM spermidine, 2.5 mM DTT, 0.3 mM amino acid mix, 1.2 mM ATP, 0.25 mM GTP and 16 mM creatine phosphate) by inserting the pipette tip down to the bottom of the well, thereby creating two distinct layers. The plate was then covered with the sealing film, and was incubated at 17°C for 18 hr without shaking. For the calcium chelating experiment, 1 mM BAPTA (1,2-bis(o-aminophenoxy)ethane-N,N,N',N'-tetraacetic acid) (Sigma-Aldrich, St Louis, MO) was added to both the translational mixture and TSB. For biotin labeling of proteins [15,20], 2 μg of biotin protein ligase (BirA, GenBank accession no. NP_0312927) produced by the wheat cell-free system and 6 μM D-biotin (Nacalai Tesque, Kyoto, Japan) were added to the bottom translational mixture.

For co-expression of the biotinylated srtA-LPETG-UBE2N and UBE2v1, the cell-free production method was slightly modified. Each translation mixture was prepared independently and pre-incubated at 26°C for 30 min, following which they were mixed, and were subsequently used in the bilayer translation reaction as described above.

### Self-cleavage purification of proteins produced by the cell-free system

Reaction mixture (6 mL) from the cell-free expression system described above was mixed with 100 μL of Ni-NTA sepharose (GE Healthcare) or 100 μL of streptavidin-sepharose (GE helthcase). The sepharose beads were pre-equilibrated with phosphate buffered saline (PBS) for 4 to 6 hours (His tag) or 30 min (BISOP) at 4°C. Sepharose bead-captured srtA-fusion proteins were collected by centrifugation (3,000 × g) and the beads were washed three times with PBS buffer. Self-cleavage purification of the target protein was performed by incubation of the beads with 100 μL of Elution buffer [20 mM Tris-HCl (pH 7.5), 5 mM Tri-Gly (Sigma-Aldrich), 5 mM CaCb, 150 mM NaCl, 1 mM DTT and 2% glycerol] for 4 hours at 16°C. The buffer-bead mixture was then transferred into a micro spin-column (GE Healthcare) and the eluted fraction was recovered by flash centrifuge (3,000 × g) at 4°C. The N-terminal end of the eluted GFP was determined by amino acid sequence analysis using the Applied Biosystems ABI 473A protein sequencer and according to the instructions provided by Applied Biosystems.

The cell-free protein production and self-cleavage purification were also carried out using an automatic robot, Protemist DTII (CellFree Sciences Co., Ltd.), basically according to manufacturer's instructions. Addition of biotinylation reagents and BAPTA were also carried out as mentioned above.

### Phosphorylation assay

The phosphorylation assay was mainly performed according to the previous published methods [13,21]. To assay for phosphorylation of the biotinylated Src by CSK, 40 μl of the reaction mixture was mixed with 15 μl of biotin magnetic beads (Promega, MI), and was washed twice with PBS buffer and once with protein kinase (PK) buffer [50 mM Tris-HCl (pH 7.6), 500 mM potassium acetate, 50 mM MgCl_2_, 0.1 mM DTT]. Beads were suspended in 10 μl PK buffer supplemented with cold ATP (for immunoblot) or [*γ*-^32^P]-ATP (for autoradiogram), and CSK purified using the BISOP method, UBE2N or commercially available GST-CSK fusion protein (purchased from Carna Biosciences Inc., Kobe) was added in the reaction mixture. The mixtures were incubated at 37°C for 30 min, following which they were boiled in the SDS-denaturing buffer and the proteins were separated on 12.5% SDS-polyacrylamide gel. Autoradiogram of Src phosphorylation was analyzed by BAS-2500 (FUJIFILM, Tokyo, Japan). For immunoblot analysis, proteins were transferred from the SDS-PAGE gel to PVDF membrane (Millipore Bedford, MA, USA) following standard procedures. The blots were then processed using the Immobilon Western detection reagents (Millipore) and antibody against phosphorylated Src (Y527) or Src (Cell Signaling Technology, Beverly, MA) according to the manufacturer's procedure. The anti-phospho-Src (Y527) antibody recognizes the phosphorylated Y530 residue in human Src.

## Abbreviations

srtA: sortase SrtA; GFP: green fluorescent protein; SGK: serum/glucocorticoid regulated kinase 1; CSK: c-src tyrosine kinase; UBE2N: ubiquitin-conjugating enzyme E2N; UBE2v1: ubiquitin-conjugating enzyme E2 variant 1; AMPKa1: 5'-AMP-activated protein kinase alpha 1 catalytic subunit; PRKAA1: protein kinase, AMP-activated: alpha 1 catalytic subunit; MAPK12: mitogen-activated protein kinase 12; p38g: p38 gamma; Pfs25: *Plasmodium falciparum *25 kDa ookinete surface antigen precursor; BAPTA 1: 2-bis(o-aminophenoxy)ethane-N,N,N',N'-tetraacetic acid; PBS: phosphate buffered saline.

## Authors' contributions

SM conceived the study and performed some of the experiments; KM and KS performed also participated in performing the experiments; YE conceived the study and supervised the work; TS conceived and designed the study, supervised the work and contributed to writing the manuscript. All authors read and approved the final manuscript.
